# Rev. J. S. Grimshaw's *Vindication of Ann Foulkes*

**Published:** 1814-01

**Authors:** J. S. Grimshaw

**Affiliations:** Vicar of Bidenham, Beds. Bedford


*To the Editor of the Medical and Physical Journal.*

SIR,

ON perusing Mr. Gibbon's representation of the case of Ann Foulkes, an impression arose in my mind that some of the facts therein stated differed very materially from the account I had received from her, *at the period of their occurrence;* and, as an inference was drawn from them prejudicial to the moral character of Ann Foulkes, I thought it an act of justice due to the poor girl, whom I have now attended upwards of four years, not without the highest approbation of her principles, to ascertain how far my recollection of facts was well founded or not. With this view I waited upon her in company with a friend, when she was minutely questioned as to the following facts, namely, *the proposed introduction of the catheter; and the circumstance recorded by Mr. Gibbon of his having patiently waited that he might personally witness the reality of the urinous vomiting.*

The following is the result of the examination, taken almost verbatim. I feel a considerable degree of reluctance in communicating the substance of it, but the complexion this controversy has taken is so decidedly hostile to the reputation of more than one character, that I have no longer the option of suppressing so important a document.

Q. What conversation passed between you and Mr. Gibbon about the catheter?—A. Mr. Gibbon said, he must use the instrument; to which I replied, No, not unless you send for Dr. Yeats.

 Q. Did

Q. Did Mr. Gibbon, on this, offer to send for Dr. Yeats?—A. No, he did not.

Upon being asked why she was so unwilling that Mr. G. should use the catheter? she replied, because he was a stranger to her, and not her doctor, and she did not wish to submit to any experiment without the knowledge of Dr. Yeats.

Q. Did Mr. Gibbon still urge the use of the catheter?—A. Yes; on his retiring from the room, he turned round, and angrily said, I insist upon doing it, and on Sunday next I will attend for that purpose. I again answered, No, sir, not unless you send for Dr. Yeats; and if he thinks it will be of any service, I am willing to submit, but not otherwise.

Q. Did Mr. Gibbon then propose sending for Dr. Yeats to perform the operation?—A. No, he never once alluded to it.

Q. Are you willing to declare this on oath?—A. I am.

Q. Was any one present besides?—A. Yes, my mother.

Q. Do you remember the facts as here stated? (addressed to the mother.)—A. Yes, I do.

Q. Will you swear to the truth of them?—A. I am perfectly willing to do so.*

Q. Did Mr. Gibbon ever see you in the act of urinous vomiting?—A. I sent for Mr. Gibbon, at his own request, once when I had taken an emetic, and had vomited twice before he came. On my not vomiting immediately in his presence, he grew impatient. I said, Stop, sir, for I think I shall vomit again; he replied he could not, and desired she would make haste. She said she could not vomit when she pleased; he observed, I have got my own patients to attend to, do you think I can wait upon you all day? and then went away.†

The whole of this examination at once confirmed the previous impressions of my own mind, *and agreed with the statement of facts given to me by herself, near the time of their occurrence, which I believe was nearly twenty months ago, before the subject had assumed the character of controversy.* I should add that other highly respectable persons have the same recollection of the above fact as myself, and can bear testimony to it, if necessary.

With respect to the quantity of provision, clothing, and

---

* In Mr. Gibbon's statement are the following words: "Upon offering to send for Dr. Yeats," &c. &c. See Medical Journal, No. 177, p. 358.

† Mr. Gibbon's words are, "I therefore waited *patiently* for more urine to make its appearance: she professed to be very sick, but none came." Ibid, page 358.

money money which it is stated she received in such abundance, I have reasons for knowing that they were not beyond the supply of common exigencies, and that periods have occurred when she has been reduced to much want.

The public must now judge, from the statements given, how far the character of the poor object in question has or has not been unjustly assailed. I cannot willingly prevail upon myself to think that Mr. Gibbon has designedly mis-stated facts: at the same time I cannot withhold my belief from the testimony of the girl, whose moral character I firmly believe to be unimpeachable; and my recollection of the statement, sanctioned as it is by others of great respectability, *at the period of the occurrence itself*, is a fact which will not escape observation. Why Dr. Yeats was not sent for, why he was not more communicated with, I am not able to comprehend; but that personality of a very unpleasant kind has unfortunately mingled itself with this controversy, every day brings along with it some painful testimony on the part of Mr. Gibbon. So far as the moral character of Ann Foulkes is concerned, if analogous cases in the history of medicine continue to be brought forward, to illustrate the possibility of urinous vomiting, the charge of want of veracity, which was founded against her, on the supposed impossibility of such an occurrence, is wholly at an end. If so, what are the sentiments which will arise in every delicate and truly honorable mind on the review of this transaction? But I forbear to expatiate on so painful a subject. My only prayer is that Mr. Gibbon may never be able to judge of the pangs inflicted by the unmerited loss of reputation, by experiencing a similar fate himself. If this be his wish, I need not suggest to him, that one safeguard to his own reputation will be not to intrench upon that of others.

I have the honor to be, Sir,

Your very obedient Servant,

J. S. GRIMSHAW,

*Bedford, Dec.* 9, 1813. *Vicar of Bidenham, Beds.*

